# Bone Tissue Response to Porous and Functionalized Titanium and Silica Based Coatings

**DOI:** 10.1371/journal.pone.0024186

**Published:** 2011-09-15

**Authors:** Amol Chaudhari, Annabel Braem, Jozef Vleugels, Johan A. Martens, Ignace Naert, Marcio Vivan Cardoso, Joke Duyck

**Affiliations:** 1 Department of Prosthetic Dentistry, BIOMAT Research Group, K. U. Leuven, Leuven, Belgium; 2 Department of Metallurgy and Materials Engineering (MTM), K. U. Leuven, Heverlee, Belgium; 3 Centre of Surface Chemistry and Catalysis, K. U. Leuven, Heverlee, Belgium; Massey University, New Zealand

## Abstract

**Background:**

Topography and presence of bio-mimetic coatings are known to improve osseointegration. The objective of this study was to evaluate the bone regeneration potential of porous and osteogenic coatings.

**Methodology:**

Six-implants [Control (CTR); porous titanium coatings (T1, T2); thickened titanium (Ti) dioxide layer (TiO_2_); Amorphous Microporous Silica (AMS) and Bio-active Glass (BAG)] were implanted randomly in tibiae of 20-New Zealand white rabbits. The animals were sacrificed after 2 or 4 weeks. The samples were analyzed histologically and histomorphometrically. In the initial bone-free areas (bone regeneration areas (BRAs)), the bone area fraction (BAF) was evaluated in the whole cavity (500 µm, BAF-500), in the implant vicinity (100 µm, BAF-100) and further away (100–500 µm, BAF-400) from the implant. Bone-to-implant contact (BIC-BAA) was measured in the areas where the implants were installed in contact to the host bone (bone adaptation areas (BAAs)) to understand and compare the bone adaptation. Mixed models were used for statistical analysis.

**Principal Findings:**

After 2 weeks, the differences in BAF-500 for different surfaces were not significant (p>0.05). After 4 weeks, a higher BAF-500 was observed for BAG than CTR. BAF-100 for AMS was higher than BAG and BAF-400 for BAG was higher than CTR and AMS. For T1 and AMS, the bone regeneration was faster in the 100-µm compared to the 400-µm zone. BIC-BAA for AMS and BAG was lower after 4 than 2 weeks. After 4 weeks, BIC-BAA for BAG was lower than AMS and CTR.

**Conclusions:**

BAG is highly osteogenic at a distance from the implant. The porous titanium coatings didn't stimulate bone regeneration but allowed bone growth into the pores. Although AMS didn't stimulate higher bone response, it has a potential of faster bone growth in the vicinity compared to further away from the surface. BIC-BAA data were inconclusive to understand the bone adaptation.

## Introduction

The concept of osseointegration of titanium (Ti) implants [1] is well known and well documented. With the help of this knowledge, an osseous interface can reproducibly be formed and maintained at the implant surface for its long term use. [2] Although implant treatments have very high success rates, [3] compromising conditions such as limited bone healing capacities (in diabetic patients, smokers) and irradiated or grafted host bone may lead to implant failure. [4] Moreover, the clinical practice is shifting from an unloaded healing period to immediate or early implant loading. [5] As implant stability is crucial for the prognosis of early or immediately loaded implants, osseointegration should be established as fast as possible. This makes that not only the quality, but also the acceleration of the osseointegration process becomes important. This leads to an increasing interest to find new possibilities for improving the quality and the speed of osseointegration, by means of physical and/or chemical modification of implant surfaces, use of mechanical loading and implant design optimization. [6]–[8]

The rationale behind changing the implant surface properties is to increase the speed of post-implantation biological events so that the osseointegration can be achieved at a faster rate. Implant surfaces that stimulate the recruitment of premature osteogenic cells, leading to a more efficient peri-implant bone formation may eventually lead to a faster osseointegration. It is, however, difficult to predict the cell behavior, even on a surface of well-known properties. [9] The *in vivo* situation is more complex and not all aspects of the *in vivo* environment are predictable. Nevertheless, the experimental evidence shows that osteoprogenitor cells have a higher affinity for particular surface topographies and functional groups, which stimulate them to form bone. [10], [11] It is therefore desirable to apply modification techniques that can produce rough and/or functionalized surfaces.

Due to the pioneering efforts of Brånemark et al., [12] Ti became a favored material for implants. Ti has a very good chemical and biological stability owing to the spontaneously formed dioxide layer (TiO_2_) at the surface. [13] The thickness of the TiO_2_ layer can be grown further by means of a technique called anodic oxidation. [14] Depending on the experimental conditions, the properties of this oxide layer can be changed in terms of chemical composition and its 3D porous structure. These surface features hold potential to improve bone growth over the implant surface. [15]

During the last three decades the use of cellular porous materials with the characteristics of high roughness (in µm range) and high porosities (above 50%) has come a long way from research to the clinical level. [16], [17] Although there are conflicts about the optimal range of topographic and bulk properties of the coatings, [18], [19] there is enough evidence that these coatings improve the mechanical interlocking at the implant-surface/bone interface due to bone tissue growth into the pores. [20] Similarly, nano-structured surfaces possess unique properties that alter cell adhesion either due to the effect of their structure on the cells directly or indirectly through initial surface protein interactions. [11] In this regard, meso-porous (2 nm≤ pore size ≤50 nm [21]) and micro-porous (2 nm≤ pore size [21]) materials prepared via sol-gel method can be used because of their biocompatibility, tunable structure and the possibility of incorporation of growth factors. [22] In the group of micro-porous materials, AMS is a sol-gel based silica material, the pores of which are suitable for incorporation of bioactive small molecules and their controlled release. [23]–[25]

Silica (SiO_2_) based bio-active glass (BAG) materials [26] are capable of bone formation at the endosseous implant surface, avoiding its fibrous encapsulation when applied in the form of a stable mechanical coating. [27] In a biological environment, different ionic components are released from such glass matrices. Released components such as Ca^2+^ and PO_4_^3−^ are known to promote osteoconduction by forming a calcium phosphate layer at the surface. [28] Another advantage is the possibility of incorporation of osteogenic agents which can improve the quality and rate of healing at the required place. [29] Thus, from the bone regeneration point of view, SiO_2_ based BAG exhibits many properties which can be exploited for a clinical benefit.

Five different surfaces were chosen and prepared for studying the tissue response in the *in vivo* rabbit model. The different surface roughness, chemical composition and functional groups of the test surfaces led to varying surface physical and chemical properties. The following surfaces were tested: 1) two porous, pure Ti coatings with a rough topography and varying internal pore structures 2) a Ti surface with a thickened dioxide layer 3) Amorphous Microporous Silica (AMS) coating and 4) a Bio-active Glass (BAG) coating. It was hypothesized that these test surfaces improve peri-implant bone formation and osseointegration compared to commercially pure Ti (cpTi) at two healing periods of 2 and 4 weeks.

## Materials and Methods

### Implant design and surface modification

Commercially pure Ti sheets (grade 2, Goodfellow, Huntingdon, UK) 1 mm in thickness were laser cut into discs (Ø 15.5 mm for the surface characterization and Ø 4 mm as implants for the *in vivo* study). After ultrasonic cleaning in acetone (Acros Organics, Geel, Belgium) and washing in excess distilled water, the discs were acid-etched in a solution containing 4 vol % HF (40%, Riedel-de haen, Belgium) and 20 vol % HNO_3_ (65%, Chemlab, Zedelgem, Belgium) at room temperature for 60 s. Finally, the Ti discs were washed again in excess distilled water and autoclave sterilized in separate inert containers. The cleaned Ti substrates represented the control group (CTR). The CTR surfaces were modified by 5 test coatings (T1, T2, TiO_2_, AMS and BAG).

Porous coatings of pure Ti were manufactured by electrophoretic deposition (EPD) of Ti hydride (TiH_2_) powder followed by dehydrogenation and sintering in vacuum. A first type of porous Ti coating (T1) was applied as described previously by Braem et al. [30] TiH_2_ powder (grade P, Chemetall GmbH, Frankfurt (M), Germany) suspension in absolute ethanol (analytical grade, Prolabo, Haasrode, Belgium) was prepared using polyethyleneimine (PEI, 50 wt% in water, Sigma, Bornem, Belgium) as a charger and binder. The suspension was consolidated through EPD, using a controlled voltage power supply (MCN 1400-50, FUG, Rosenheim, Germany). The Ti substrate was vertically placed in the deposition cell and coupled as cathod. Finally, the samples were dehydrogenated at 650°C and sintered at 850°C by resistive heating in vacuum (10^−6^ mbar). For the second porous Ti coating (T2), TiH_2_ powder stabilized emulsion was prepared similar to the procedure as described by Neirinck et al. for titanium powder deposition, [31] while using cyclohexane (Prolabo, Haasrode, Belgium) instead of paraffin oil as the dispersed phase. After adding a TiH_2_ suspension (as used for T1), this emulsion was consolidated through EPD and dehydrogenated and sintered as indicated. The application of a particle stabilized emulsion results in an interconnected pore structure similar to T1, but with additional spherical pores included. The TiO_2_ layer on the CTR surface was thickened by anodic oxidation in 1 M H_3_PO_4_ (85%, Riedel-de Haen, Belgium) using a controlled voltage power supply (MCN 1400-50, FUG, Rosenheim, Germany).

AMS was prepared according to Maier et al. [32] Appropriate amounts of tetraethoxysilane (TEOS) (98%, Acros Organics, Geel, Belgium), absolute ethanol (VWR, Haasrode, Belgium) and HCl (37%, Chemlab, Zedelgem, Belgium) were mixed to obtain a colloidal sol. The molar ratios of TEOS:ethanol:HCl:H_2_O were 1∶3∶1.74∶6. A sol was obtained by stirring the mixture for 1 h (250 rpm, Variomag Multipoint 15, Daytona Beach, USA). The sol was then diluted 5 fold in absolute ethanol. The diluted sol was immediately spin-coated on CTR surfaces. Spin-coating was performed using an Erichsen device at 1500 rpm for 60 s. The coatings were dried at room temperature. Finally, the calcination was carried out by heating up to 65°C and after 5 h heated further till the final temperature of 300°C. A heating rate of 0.1°C min^−1^ was maintained throughout the heating procedure. After 5 h at the final temperature, the coatings were cooled to ambient temperature.

BAG coatings were applied on CTR surfaces that were previously treated by anodic oxidation as described above. This was done by dipping the substrates during 120 s in a 5 wt% absolute ethanol based suspension of melt-derived BAG powder, with a nominal composition of 50.1 wt% SiO_2_, 25.2 wt% CaO, 20.1 wt% Na_2_O & 4.6 wt% P_2_O_5_ and an average particle size of 1 µm. The samples were allowed to dry for 24 h and sintered at 800°C by resistive heating in vacuum (10^−6^ mbar). After sintering, 6 surfaces were submerged in cell culture medium for 24 h to evaluate pH changes of the medium.

### Characterization

#### Surface topography and bonding strength of the coatings

Quantitative three-dimensional topographical analysis was performed by the calculation of average roughness (S_a_, amplitude parameter), texture aspect ratio (S_tr_, spatial parameter) and developed interfacial area ratio (S_dr_, hybrid parameter). The analysis was carried out by MountainsMap® Premium software (Digital Surf sarl, Besancon, France). Three samples were analyzed per group using a scanning white light interferometer (WLI, Wyko NT 3300; Veeco Metrology Inc., Tucson, USA), employing a vertical scanning interferometer technique. A total of four equidistant locations were measured per disc, which resulted in 12 measurements per group. After roughness evaluation, the same surfaces were qualitatively analyzed using a field-emission-gun Scanning Electron Microscope (Feg-SEM, XL 30, Phillips, Eindhoven, The Netherlands). For understanding bulk characteristics of the porous metal coatings (T1 & T2) and BAG coatings, metallographic cross-sections of the coatings were prepared and examined using SEM. Elemental analysis was also carried out for BAG cross sections before and after implantation using SEM with associated energy dispersive X-ray spectroscopy (SEM-EDS, EDAX). The tensile adhesion bond strength of the coatings on the Ti substrate was evaluated with an adapted version of ASTM 1147 using an INSTRON 4467 with a load cell of 30 kN at a crosshead speed of 2.5 mm/min. [30] Such characterization was carried out for T1, T2 and BAG surfaces. A specimen assembly of a coated Ti substrate (Ø 15.5 mm) and an uncoated counter body (titanium rod, Ø 10 mm), glued together with FM 1000 adhesive film (Cytec, NJ, USA) was subjected to a tensile load perpendicular to the coating surface.

### Ethics Statement

The research protocol was approved by the ethical committee for laboratory animal research of the Catholic University of Leuven and was performed according to the Belgian animal welfare regulations and guidelines. (Approval ID: P122/2008)

### Surgical procedure

**Figure 1:**
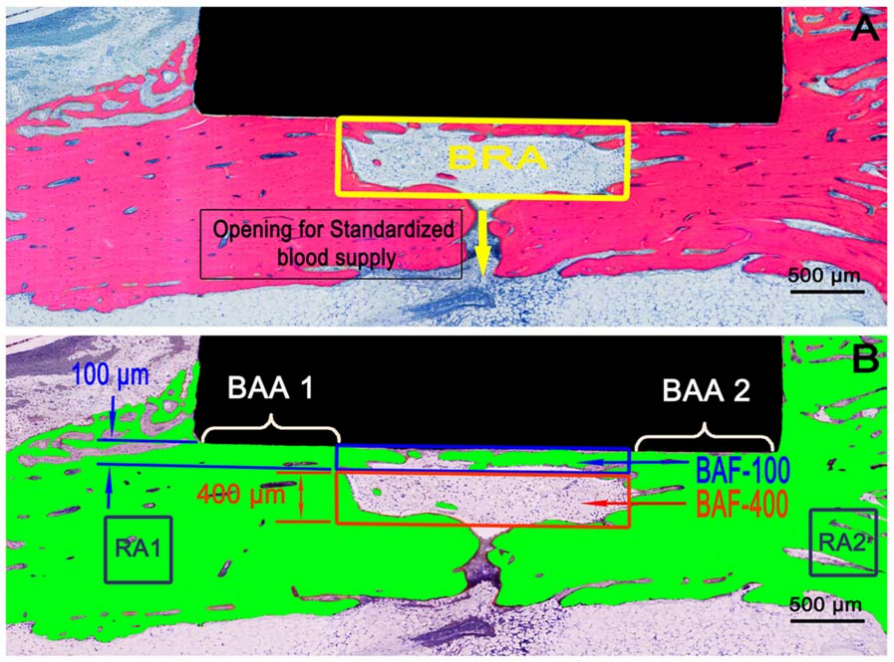
Representative histological section, retrieved after 4 weeks of healing. (A) Scanned image of the histological section. (B) The same image after being digitally processed for histomorphometrical analyses. Four well-defined areas of interest are delimitated: The bone regeneration area (BRA) divided into 100 and 400 µm zones for the measurement of bone area fraction parameters (BAF-100 and BAF-400 respectively) and two reference areas (RA's) positioned in non-affected cortical bone, at each side of the double-stepped cavity. Additionally, two bone adaptation areas (BAA's), on each side of the BRA were used to measure bone-to-implant contact (BIC-BAA).

Twenty 6-month old New Zealand white rabbits with an average weight of 3.91±0.29 kg were used in this study. All rabbits were kept in quarantine for 4 weeks before the surgery. Surgeries were performed under aseptic conditions. First, the animals were pre-medicated with an intramuscular neuroleptic analgesic (Vexylan®, 1 mg/kg, CEVA, Brussels, Belgium) and with an intramuscular anaesthetic (Ketamine 1000® 15 mg/kg, CEVA, Brussels, Belgium). During the surgery, anesthesia was maintained using isoflurane, USP (Halocarbon, River Edge, USA). After applying local anesthetic (Lignospan, Septodond, Cedex, France) subcutaneously at the surgical site, a longitudinal incision was made on the medial side of the proximal tibia and both soft tissues and periosteum were mobilized. Three cavities were prepared exclusively in the cortical bone of each proximal tibia diaphysis using a custom-made rotary instrument and a low-speed hand piece under copious saline irrigation. This cutting tool was specially designed to create a circular cavity of 4 mm diameter containing a smaller inner cavity of 2 mm diameter and a depth of 0.5 mm. The base of the cavity was perforated at the centre with a 0.5 mm drill to ensure standardized blood supply during healing (Figure 1A). Sterilized implants were placed randomly in two tibiae. The outer cavity was just deep enough to create a step where the implants could be positioned. These samples were then covered with a Ti osteosynthesis plate and fixed on the cortical bone by means of two Ti osteosynthesis screws (Nobel Biocare, Goteborg, Sweden). This ensured a stable initial fixation of the implants during the healing period. Skin and fascial layers were sutured separately. Post operatively, the animals were medicated with intramuscular buprenorfin as analgesic (Temgesic® 0.05 mg/kg body weight, Schering-Plough NV, Brussels, Belgium) and intramuscular antibiotic (penicillin) for 3 days at a dose of 300.000 IU daily (Kela NV, Hoogstraten, Belgium). Each animal received all experimental and control implants. The surgical site was allowed to heal for 2 and 4 weeks in 10 rabbits for each healing period. After the healing periods, the animals were sacrificed with a 0.1 ml/kg body weight intravenous injection of an embutramide-mebenzoniumjodide-tetracaine HCl solution (T61®, Intervet, Mechelen, Belgium).

The double-stepped cavity allowed the identification of two well-defined areas of interest (Figure 1A, 1B) in the histological section. The bone regeneration area (BRA) coincided with the limits of the inner cavity and thus represented a region without pre-existing bone-to-implant contact (BIC) with a width of 2 mm. Secondly, the area located at the periphery of the implant disc was named bone adaptation area (BAA) and represented a region of initial direct contact between bone and implant. Two reference areas (RA's) were determined (Figure 1B) aside each bone adaptation area and outside the disc area to calibrate the bone's natural degree of porosity. The role of these RA's was to provide information on the bone fraction of the bone which was not affected by the trauma caused by the surgery. By considering the bone area fraction (BAF) of RA's for the final measurement of the bone area fraction parameters for bone regeneration analysis, it was ensured that the inherent variability in bone density of different animals and even distinct areas of cortical bone were taken into account.

### Histological and histomorphometrical analysis

To evaluate the tissue growth into the inner cavity and the bone response around the implant surface, the specimens were prepared for histological analysis. Ti discs and surrounding tissues were gathered en bloc, fixed in a CaCO_3_ buffered formalin solution for 3 days and dehydrated in an ascending series of ethanol concentrations over 15 days. Embedding was performed by infiltration and polymerization of methylmetacrylate solution containing 0.018% benzoylperoxide as catalyst over 14 days. Samples were then sectioned transversely perpendicular to the implant using a diamond saw (Leica SP 1600, Leica Microsystems, Nussloch, Germany). The most central section of each implant was reduced to a final thickness of approximately 30 µm using a micro-grinding system (Exakt 400 CS, Exakt, Norderstedt, Germany). The sections were stained with a combination of Stevenel's blue and Von Gieson's picrofuchsin. Histological examinations were performed using a light microscope (Laborlux, Leica, Wetzlar, Germany) at a magnification of 40×, 100× and 400×. Images were captured using a high sensitivity color video camera (JVC TK-1280E, Ibaraki-ken, Japan) mounted on the light microscope and the assessments of the histomorphometrical proportions were performed using a commercially available image analysis software (Axiovision 4.0, Zeiss, Gottingen, Germany) and a customized script particularly programmed for the needs of the semi-automatic analysis.

All areas of interest mentioned in this study were semi-automatically demarcated in a reproducible manner using customized scripts made and ran in the image analysis software. Finally, the following variables were recorded for each histological section: The ***Bone area fraction*** (BAF-500, BAF-100 and BAF-400, in %) was calculated as the percentage of the BRA occupied by bone trabeculae (BT) proportional to the bone fraction of the reference areas (RA1 and RA2). The BAF of the whole cavity area was designated as BAF-500. The BAF measurement in the area within 100 µm from the implant was indicated as BAF-100. BAF-400 on the other hand, refers to the BAF measurement in the region of 100 to 500 µm away from the implant. (Figure 1B) The BAF's were calculated according to the following formulas:
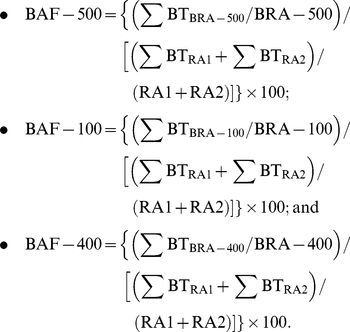



The ***bone-to-implant contact ratio in the BAA*** (BIC-BAA, in %) was calculated by the sum of the regions where bone was in direct contact to the implant (BIC) in proportion to the total length (L) of the Ti surface under analysis. 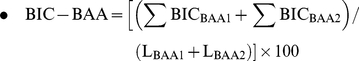



### Statistical analyses

The statistical analyses were carried out using the SAS 9.2 software package (SAS Institute Inc.). A mixed model was used since it takes into account the clustered nature of the data. As fixed effects, we have the implant and the interaction between the implant and the healing time. Since, all implants of each group were placed in the same rabbit; the animal was used as a random effect. Diagnostic plots were used to check normality and homogenity of the residuals of the model. By using SAS' estimate option of proc mixed, the differences between the measurements of the implants at each healing period were calculated and the evolution over time (going from 2 to 4 weeks) of the measurements for each implant. By using the corresponding p-value, the differences were considered as significant when the 0.05 threshold was exceeded. Parameters BAF-100 and BAF-400 were statistically compared. For further analysis, the difference in BAF in the area close (BAF-100) vs. further away (BAF-400) from the implant was found out and the change of these differences between BAF-100 and BAF-400 over time (going from 2 to 4 weeks) indicate the speed with which the BAF is changing in the respective areas. For the roughness comparison of the test and the CTR groups, normal distribution of the obtained data and the homogeneity of the variances were checked and an unpaired student's t-test was used to identify statistical differences among the different treatments in each group of surfaces at a significance level of 2%.

## Results

### Surface topography and bonding strength of the coatings

**Figure 2:**
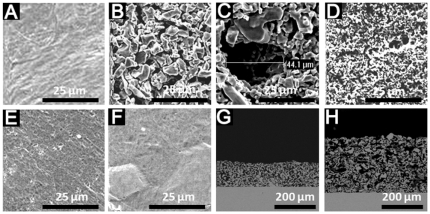
SEM images for experimental and control implants. (A) CTR, (B) T1, (C) T2, (D) BAG, (E) TiO_2_ and (F) AMS. Images (G) and (H) represent the cross-sectional views of the thick porous Ti coatings T1 and T2 (as shown by figure (B) and (C)) respectively.

**Figure 3:**
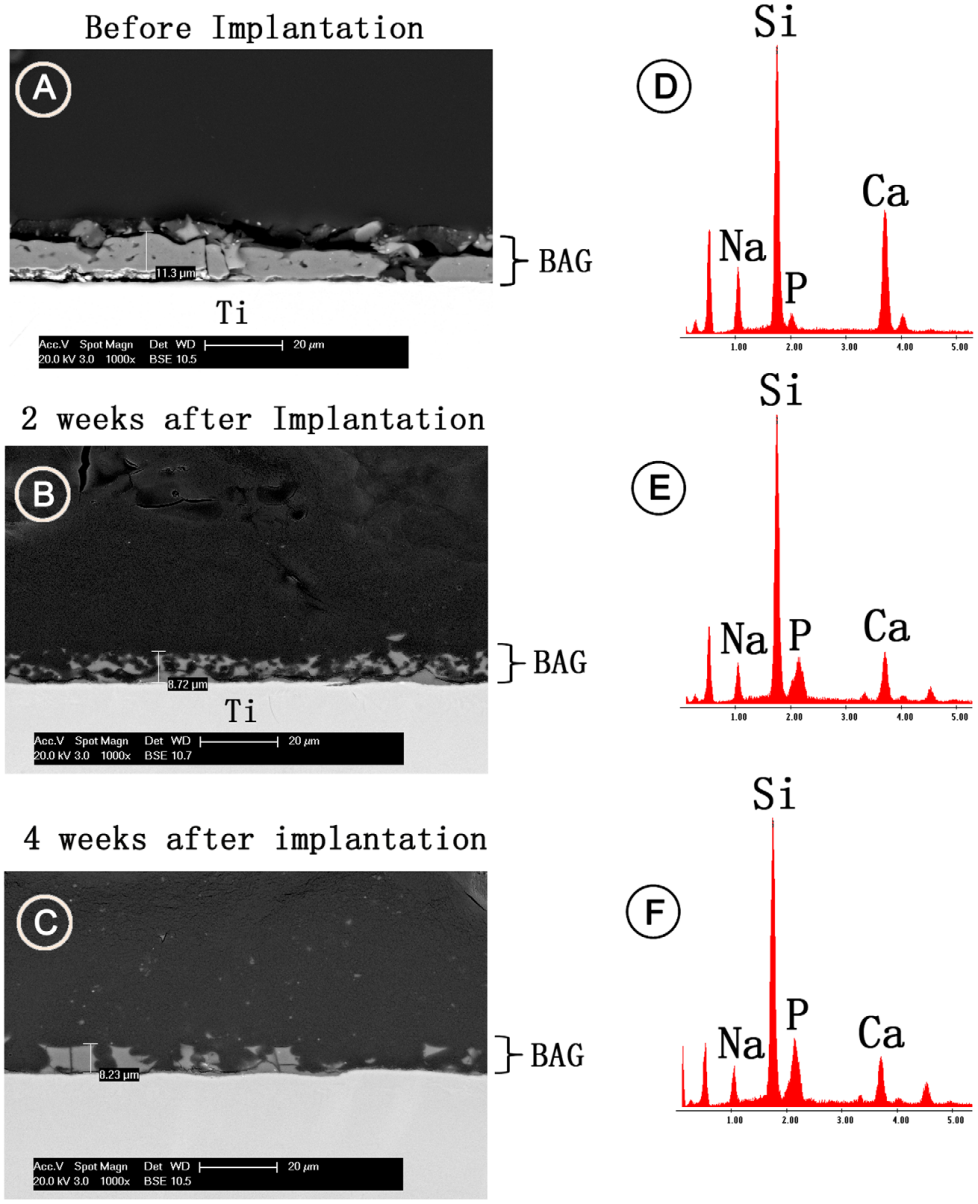
SEM images for BAG coatings in cross-sectional view. (A) before implantation, (B) after 2 weeks and (C) after 4 weeks of implantation. Images (D), (E) and (F) indicate the corresponding single point EDS analysis.

Compared to the CTR surface (Figure 2A), the qualitative surface analysis by SEM indicated that the porous coatings T1, T2 and BAG (Figure 2B, 2C, 2D) coatings gave rise to a rough surface. No apparent change in the surface morphology was observed for the TiO_2_ and AMS surfaces (Figure 2E, 2F). The cross-sectional view of the porous Ti coatings, T1 and T2 displays a very well connected porous structure which differs only in the presence of additional spherical holes of ca. 50 µm in case of T2. (Figure 2G, 2H) The approximate thicknesses of T1, T2 and BAG coating were 87 µm, 183 µm and 10 µm respectively as indicated by cross-sectional SEM images. (Figure 2G, 2H, Figure 3A) The BAG coatings almost retain their thickness after 2 and 4 weeks of implantation. (Figure 3A, 3B, 3C) But the images also indicate dissolution of the coating after the healing periods. The single point EDS analysis (Figure 3D, 3E, 3F) indicates presence of Ca and P in the BAG coating even after 4 weeks of implantation. The pH of the medium in which the BAG surfaces were submerged for 24 h was 8.2±0.04. The changes in the S_a_ value after the application of T1, T2 and BAG coatings were observed when compared to the CTR surface (P<0.02). (Table 1) T1 and T2 also differed mutually in their S_a_ values (P<0.02). The TiO_2_ surface had a similar S_a_ value as the CTR surface (P>0.02). Even though the qualitative analysis by SEM indicate no change in the surface morphology due to application of AMS coating, (Figure 2F) the roughness (Table 1) was slightly increased (P<0.02). The S_tr_ values for T1, T2 and BAG surfaces (Table 1) were close to 1 while for AMS was close to 0. For CTR and TiO_2_ the S_tr_ values were close to 0.5. The change in the surface area as measured by S_dr_ (Table 1) was higher (P<0.02) for T1, T2, AMS and BAG compared to CTR and TiO_2_. The average bonding strength for the coatings T1, T2 and BAG was 47.5±6.1 MPa [30], 28.8±7.2 MPa and 27.4±2.8 MPa respectively.

**Table 1:** Surface analysis results.

	CTR	TiO2	T1	T2	AMS	BAG
**S_a_ [µm]**	0.29±0.02	0.31±0.03	7.86±0.73	9.06±0.71	0.61±0.04	3.25±0.44
**S_tr_**	0.54±0.08	0.53±0.16	0.70±0.10	0.72±0.11	0.32±0.20	0.79±0.08
**S_dr_ [%]**	0.35±0.04	0.37±0.03	102.50±21.70	188.98±23.60	0.91±0.12	97.23±26.09

S_a_ - arithmetic mean of the absolute values of the surface departures from the mean plane.

S_tr_ - measure of spatial isotropy or directionality of the surface texture.

S_dr_ - percentage of additional surface area due to the surface modification as compared to same size of the measurement region of an ideal plane.

### Histological findings

**Figure 4:**
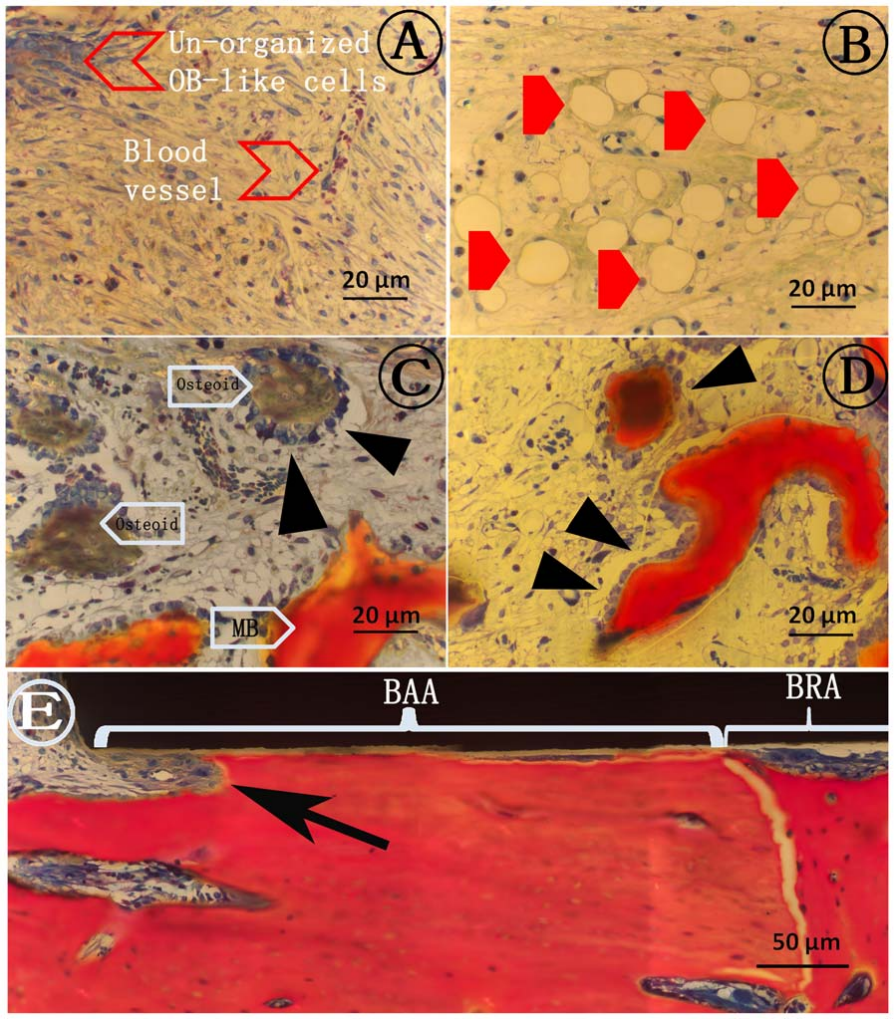
Histological analyses of the stained sections. A, B, C and D show representative aspects of bone formation inside bone regeneration areas (BRAs). (A), (B) BRA filled with non-organized soft tissue, a dense concentration of blood cells and adipocytes (filled red arrows) after 2-weeks. (C) Osteoid with osteoblast lining (black triangles) depicting a very active bone regeneration process, observed after both healing periods. (D) Later stage of bone formation (4 weeks) with a well-organized tissue including the presence of trabecular mineralized bone (MB) connected to osteoid. (E) Partial bone/implant interface in the bone adaptation area (BAA), including part of the BRA. Bone remodeling is visible at the interface (arrow).

**Figure 5:**
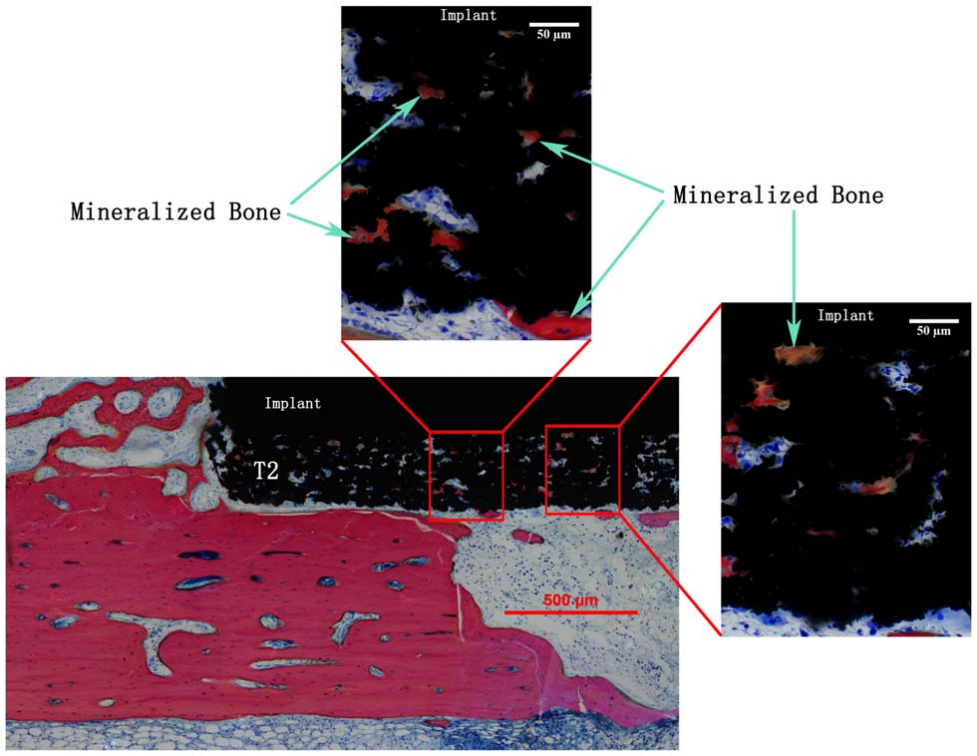
Bone growth into the pores of T2 coating after 4 weeks of healing. A similar phenomenon was observed for T1 surfaces after both the healing periods. The cells and the mineralized bone can be seen deep into the coating (up to the Ti substrate).

For both healing periods, newly formed tissue comprised of bone marrow, bone trabeculae and connective soft tissue could be observed in the regeneration area (Figure 1A). More bone formation after 4-week healing period was observed compared to 2 weeks which was also confirmed by the histomorphometrical analyses. Careful observation of the histological sections showed no signs of inflammatory response and infection. After 2 weeks of healing, blood vessels, non-organized osteoblast like cells and adipocytes could be observed in the regeneration area. (Figure 4A, 4B) Osteoid (Figure 4C) could be detected after both healing periods but its presence was more pronounced after 2 weeks than after 4 weeks of healing. Osteoblasts were lined up along the edges of the osteoid and mineralized bone (MB) indicating very active bone formation (Figure 4C, 4D). Bone resorption (Figure 4E) at the implant/bone interface in the BAA region could be observed at both healing periods though more pronounced after 4 weeks especially for AMS and BAG surfaces. Light microscope images of the histological sections after 2 and 4 weeks indicate presence of bone tissue in the pores of both T1 and T2 coatings. An example of bone growth in the pores of a T2 coating after 2 weeks of healing is shown in figure 5.

### Histomorphometrical findings

**Figure 6:**
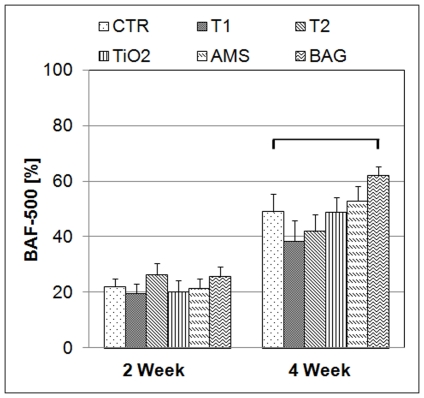
Bone area fraction (BAF) comparison for the whole cavity. The results are the mean values (bars) with standard errors of the mean after 2 and 4 weeks of healing. BAF was measured for the whole cavity (BAF-500). The horizontal line indicates a statistically significant difference (P<0.05). Significant differences for all groups are not shown for the comparison between 2 and 4 weeks.

**Figure 7:**
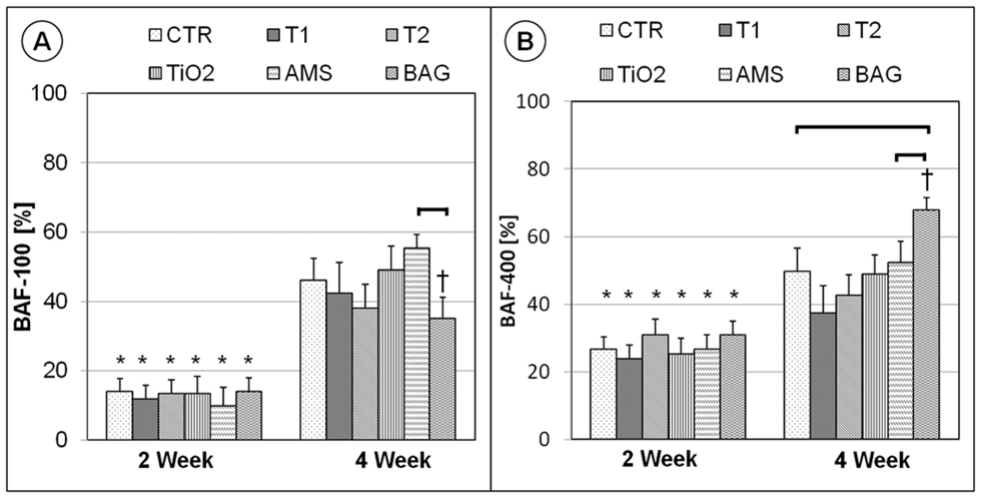
Bone area fraction (BAF) comparison for the 100- and 400-µm zones. The results are the mean values (bars) with standard errors of the mean. The horizontal lines indicate statistically significant difference. (P<0.05) The * and † indicate differences between the two parameters BAF-100 and BAF-400 (P<0.05) for the same implant and healing period. Significant differences for the comparison of 2- and 4-week results are not indicated.

**Figure 8:**
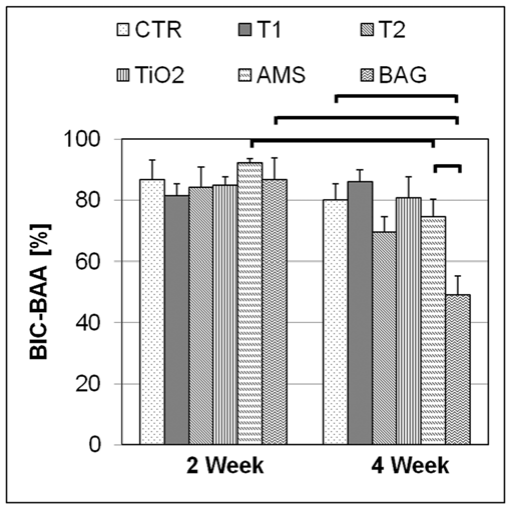
Bone-to-implant contact comparison in the bone adaptation area (BIC-BAA). The results are the mean values (bars) with standard errors of the mean. The horizontal lines indicate statistically significant difference. (P<0.05).

BAF-500 results are graphically represented in figure 6. A statistically significant difference (P<0.05) was observed for all the groups when 2- and 4-week results were compared. In the 4-week group, a statistically significant difference (P<0.05) could be observed for the BAG surface (61.8±3.2%) when compared to the CTR (49.0±6.3%). No differences were observed for other groups. After 2 weeks, all surfaces indicate significant differences when BAF-100 and BAF-400 were compared. (Figure 7A, 7B) After 4 weeks BAF-400 was higher than BAF-100 only for BAG. (Figure 7A, 7B) For the BAF parameters, no significant differences could be observed among the test groups and the CTR in the 2-week healing period. After 4 weeks, BAF-100 for AMS (55.2±4.2%) was significantly higher (P<0.05) compared to BAG (34.9±6.2%) and BAF-400 for BAG (67.9±3.7%) was higher (P<0.05) than the CTR (49.8±6.9%) and AMS (52.3±6.3%). For T1 and AMS, the change in the differences in BAF-100 and BAF-400 at 4 and 2 weeks indicate faster bone regeneration in the area close (100-µm zone) to the implant compared to the area away (400-µm zone) from the implant. The results of bone-to-implant contact in the bone adaptation area (BIC-BAA) are shown in figure 8. A statistically significant difference can be observed for AMS and BAG when the results of the 2- and 4-week were compared. After 4 weeks, BAG results were significantly lower than AMS and the CTR group. For the implant groups T1, T2, TiO_2_ and AMS no difference was observed when compared to the CTR for both healing periods.

## Discussion

For bone regeneration applications, the surface of the bulk material is an important entity, as any foreign material interacts with its biological environment through its surface. In literature, different modification paths were used by various researchers to alter the surface properties of the implants for the desired outcome. [10], [11] Other than the non-biological modification of implants, [33] surface topography adjustments and functionalization are important strategies. Surface topographies (mm, µm & nm scale) and their combination have shown to improve cell adhesion and differentiation in the osteogenic lineage. [10], [34], [35] Owing to the high surface area, such surfaces also boost the primary stability of the implant with the virtue of mechanical interlocking at the implant/bone interface. [10], [11] Porous Ti coatings (T1 & T2), AMS and BAG surfaces used in this work fall into the category of rough surfaces.

The porous and rough surface coatings (T1 and T2) did not show any improvement in the bone regeneration quality when compared to the CTR (Figure 6). As mentioned earlier, the surface morphology of T1 and T2 resembles that of other metal porous coatings. [16], [17] But the lack of consistency in the properties of such porous coatings and their corresponding results make it difficult to understand the influential property of porous metal surfaces for bone regeneration. [18], [19], [36]-[38] The three dimensional structure of the T1 and T2 coatings differ in their internal structure. The average pore size and porosity for T1 is 20 µm and 50% respectively. The respective values for T2 are 50 µm and 65%. The porosity and the cross-sectional views of T1 and T2 coatings (Figure 2G, 2H) suggest the interconnection of the pores in the bulk of the coating. It was hypothesized that the bone tissue will respond differently to these differences in the internal structure of the porous coatings which may lead to possible differences in cell delivery and mass transport required for bone regeneration to the bulk of the coating thereby leading to the bone/implant interlocking. [20] Although the histomorphometrical analysis indicates no differences in the bone regeneration capacity of the two surfaces (Figure 6), these porous structures support cell transport and bone regeneration inside the pores (Figure 5). It is interesting to observe the newly formed mineralized bone tissue was formed deep into the coating. This also reveals the interconnectivity of the pores throughout the coating.

Silica surfaces produced by the sol-gel method can improve cell adhesion and are therefore capable of faster osseointegration. [26] Such surfaces are characterized by the surface nano structure and functionalization can be imparted to these surfaces for biomedical applications. [39]–[41] In an attempt to produce a thin SiO_2_ coating similar to mesoporous SiO_2_ materials, AMS was applied to implant surfaces to study its bone stimulating potential. The application of a thin AMS coating prepared by sol-gel method slightly changed the roughness, texture aspect ratio and interfacial area ratio of the CTR surface. But these changes in the surface properties did not affect the bone regeneration capacity of AMS compared to CTR (Figure 6). The faster increase of bone in the vicinity of the implant (BAF-100) compared to further away (BAF-400) after 4 weeks of healing, indicate a positive effect of the AMS surface on bone growth close to the implant.

The bone regeneration potential of BAG surfaces is due to its degradation ionic products. [27] Comparing BAF-100 and BAF-400 (Figure 7A, 7B) after 4 weeks, BAG does not show an osteogenic effect near the implant surface. However, BAG-like surfaces are known to promote osteoconduction by apatite formation due to release of ionic components such as Ca^2+^ and PO_4_^3−^. [26] But the release of cations (Ca^2+^ and Na^+^) in the physiological situation also increases the pH of its surrounding, along with the induction of bone formation. [26], [42] It is known that the osteoblasts prefer slightly increased pH (7.8) for proliferation [43], [44] but drastic changes in the surrounding pH can inhibit osteoblast activity and cause cell necrosis and apoptosis. [43] The *in vitro* experiment to find out the changes in the pH in the surrounding of BAG surfaces indicated an increase in the pH beyond 8. Moreover, the degradation mechanism of BAG in the *in vivo* environment can be more severe compared to the *in vitro* tests carried out in this study because of the more active biological environment and the limited space for the degradation products. This increased accumulation of degradation products is likely to additional increase in the pH. As the BAG surface was not pre-treated for apatite-like structure formation before implantation, [45] the sudden increase in the alkalinity caused by the release of cations might have inhibited the osteoblast proliferation on the implant surface. But the more pronounced bone formation in the area away from the implant surface (400-µm zone) compared to the area in the implant vicinity (100-µm zone) indicates the osteogenic effect of BAG. The presence of the BAG coating (Figure 3C) on the Ti surface even after 4 weeks of healing suggests that the bone regeneration effect indeed was due to the coating. The exact mechanism which prevented bone growth on the BAG surface is unraveled in this study and therefore needs further investigation. BAG coating was made on a thickened TiO_2_ layer for better adhesion of the coating. Similar bone regeneration by CTR and TiO_2_ surfaces indicate no adverse effect of the thickened TiO_2_ layer on osteogenesis.

The presence of orthopedic and oral implants in close contact with the host tissues increases the rate of bone remodeling which is strongly dominated by osteoclast activity during the early stages of healing. [46] The faster inception of biological integration of the implants by earlier and faster bone regeneration is desirable to avoid the possible instability of the implant due to bone remodeling. Ideally, bone formation should take place to the same extent as the interfacial bone resorption in order to optimize the changeover from the primary mechanical stability to the secondary biological stability, thereby ensuring sufficient bone-to-implant contact at all times during the healing process and thereafter. This would limit the risk of interfacial micro-motion in case of early loaded implants. After implant insertion, the bone undergoes remodeling which continues at least until 4 weeks. [47] The observed reduction in the BIC-BAA values for AMS and BAG over a period of 4 weeks (Figure 8) suggests an increased bone resorption. The lower BIC-BAA for BAG compared to AMS suggests the higher bone resorption by the former surface. As a high remodeling activity was observed even after 4 weeks of healing, this study cannot conclude on the eventual effect of BAG on implant osseointegration. Longer healing observations are required for complete comprehension.

In summary, application of BAG on an implant surface showed a superior bone regeneration capacity in the cavity suggesting its osteogenic potential. Despite better results in terms of bone regeneration, BAG does not support osteoconduction. Further modification of the BAG surface properties is desirable for better and complete osseointegration of the implants. To achieve the desired effects, controlled release of BAG ionic products and dissolution of the coating should be taken into consideration. The later is important for avoiding excess inflammatory reaction which may cause callus formation. Further modification of biocompatible T1 and T2 surfaces is necessary to overcome their limited bone response results (similar to the CTR) despite having good surface topographic properties. Even though AMS fell short to improve the bone response, it did not hinder bone formation as the results were similar to the CTR surface. The possibility to use AMS in combination with other porous metal coatings and as a delivery system for drug molecules [48] and implantable biomaterial containing tissue inducing substances [49] need to be explored similar to other zeolite surfaces.

## Acknowledgments

The authors also would like to thank Ivan Laermans, Rosita Kinnart, Ann Lissens and Luc Hendrickx for their indispensable technical assistance.
